# 1,8-cineole prevents UVB-induced skin carcinogenesis by targeting the aryl hydrocarbon receptor

**DOI:** 10.18632/oncotarget.22519

**Published:** 2017-11-20

**Authors:** Jangho Lee, Su Jeong Ha, Joon Park, Yong Ho Kim, Nam Hyouck Lee, Young Eon Kim, Yoonsook Kim, Kyung-Mo Song, Sung Keun Jung

**Affiliations:** ^1^ Department of Food Biotechnology, Korea University of Science and Technology, Daejeon 34113, Republic of Korea; ^2^ Division of Functional Food Research, Korea Food Research Institute, Gyeonggi-do 13539, Republic of Korea; ^3^ Department of Agricultural Biotechnology, Seoul National University, Seoul 151-921, Republic of Korea; ^4^ Department of Food Bioscience and Technology, Korea University, Seoul 02841, Republic of Korea

**Keywords:** 1,8-cineole, aryl hydrocarbon receptor, skin cancer, cyclooxygenase-2, drug affinity responsive target stability

## Abstract

1,8-cineole is a natural monoterpene cyclic ether present in *Eucalyptus*, and has been reported to exhibit anti-inflammatory and antioxidant effects. However, the preventive effect of 1,8-cineole on skin carcinogenesis and the molecular mechanism of action responsible remains unknown. In the present study, we investigated the effect of 1,8-cineole on UVB-induced skin carcinogenesis. 1,8-cineole inhibited UVB-induced cyclooxygenase-2 (COX-2) protein and mRNA expression and prostaglandin E_2_ (PGE_2_) generation in HaCaT cells. 1,8-cineole also inhibited phosphorylation of extracellular signal-regulated kinase (ERK) 1/2, and phosphorylation of its upstream kinases, c-Src and epidermal growth factor receptor (EGFR). Quantitative real-time RT-PCR (qRT-PCR) and drug affinity responsive target stability (DARTS) assay results showed that 1,8-cineole suppressed UVB-induced expression of a target gene of the aryl hydrocarbon receptor (AhR), *cyp1a1*, and directly binds to AhR. Knockdown of AhR suppressed COX-2 expression as well as phosphorylation of ERK1/2 in HaCaT cells. Furthermore, topical treatment of 1,8-cineole on mouse skin delayed tumor incidence and reduced tumor numbers, while inhibiting COX-2 expression *in vivo*. Taken together, these results suggest that 1,8-cineole is a potent chemopreventive agent that inhibits UVB-induced COX-2 expression by targeting AhR to suppress UVB-induced skin carcinogenesis.

## INTRODUCTION

Skin cancer is the most common cancer type diagnosed in Caucasians, and its incidence is increasing annually [1, 2]. The treatment of skin cancer represents a substantial medical burden, which is estimated to cost approximately $500-700 million per year, with a higher incidence in Australia, USA and Germany [2, 3]. Ultraviolet light (UV), particularly UVB (290-320 nm) exposure is known to be a primary cause of skin cancer. UVB irradiation induces cyclooxygenase-2 (COX-2) expression and increased COX-2 levels are associated with the development of various cancer types including skin cancer [4, 5]. Exposure to UVB activates various signaling intermediates including MAPK, PI3K/Akt and AhR, via which COX-2 expression is regulated [6]. Multiple lines of evidence have shown that targeting these signaling pathways can be an effective strategy to suppress UVB-induced COX-2 expression and skin cancer [7].

The Aryl hydrocarbon receptor (AhR) is a ligand-activated transcription factor that forms a complex with Hsp90, XAP2, and c-Src. Environmental pollutants such as 2,3,7,8-tetrachlorodibenzodioxin (TCDD) and benzo[*a*]pyrene act as AhR agonists [8]. Upon ligand binding, AhR translocates from the cytosol to the nucleus and induces the transcription of cytochrome P450 enzymes like CYP1A1, which is associated with the metabolism of exogenous pollutants [9]. The activation of AhR signaling is therefore a prominent event during inflammation and carcinogenesis [10]. A previous study has reported that AhR knockout (AhR^−/−^) mice exhibit resistance to benzo[*a*]pyrene-induced skin carcinogenesis [11]. UVB irradiation also activates AhR and subsequently induces COX-2 expression [12]. However, the effect of AhR inhibitors on the prevention of skin carcinogenesis has not been investigated in detail.

Chemoprevention refers to the targeting and suppression of reversible tumor promotion before tumor progression occurs to a significant extent [7], preventing benign tumors from converting into malignant (invasive and metastatic) tumors [13]. Accumulating evidence has shown that some phytochemicals effectively prevent the promotion of various type of tumors [14]. 1,8-cineole (also known as eucalyptol) is a natural monoterpene cyclic ether abundantly present in various plant species including *Eucalyptus*, *Rosmarinus*, and *Salvia*. 1,8-cineole has been reported to exhibit anti-oxidative [15] and anti-inflammatory [16–18] effects. Some studies have shown that 1,8-cineole reduces the spread of infectious bacteria [19] and amyloid- β-induced COX-2 expression [20]. In addition, we have observed that *Curcuma zedoria* is an abundant source of 1,8-cineole that inhibits UVB-induced COX-2 expression (unpublished data). Although 1,8-cineole has promising potential to prevent skin carcinogenesis by regulating COX-2 expression, the extent of its preventive effect and the underlying mechanism responsible has remained unknown.

## RESULTS

### 1,8-cineole inhibits UVB-induced COX-2 expression and PGE_2_ generation in HaCaT cells

**Figure 1:**
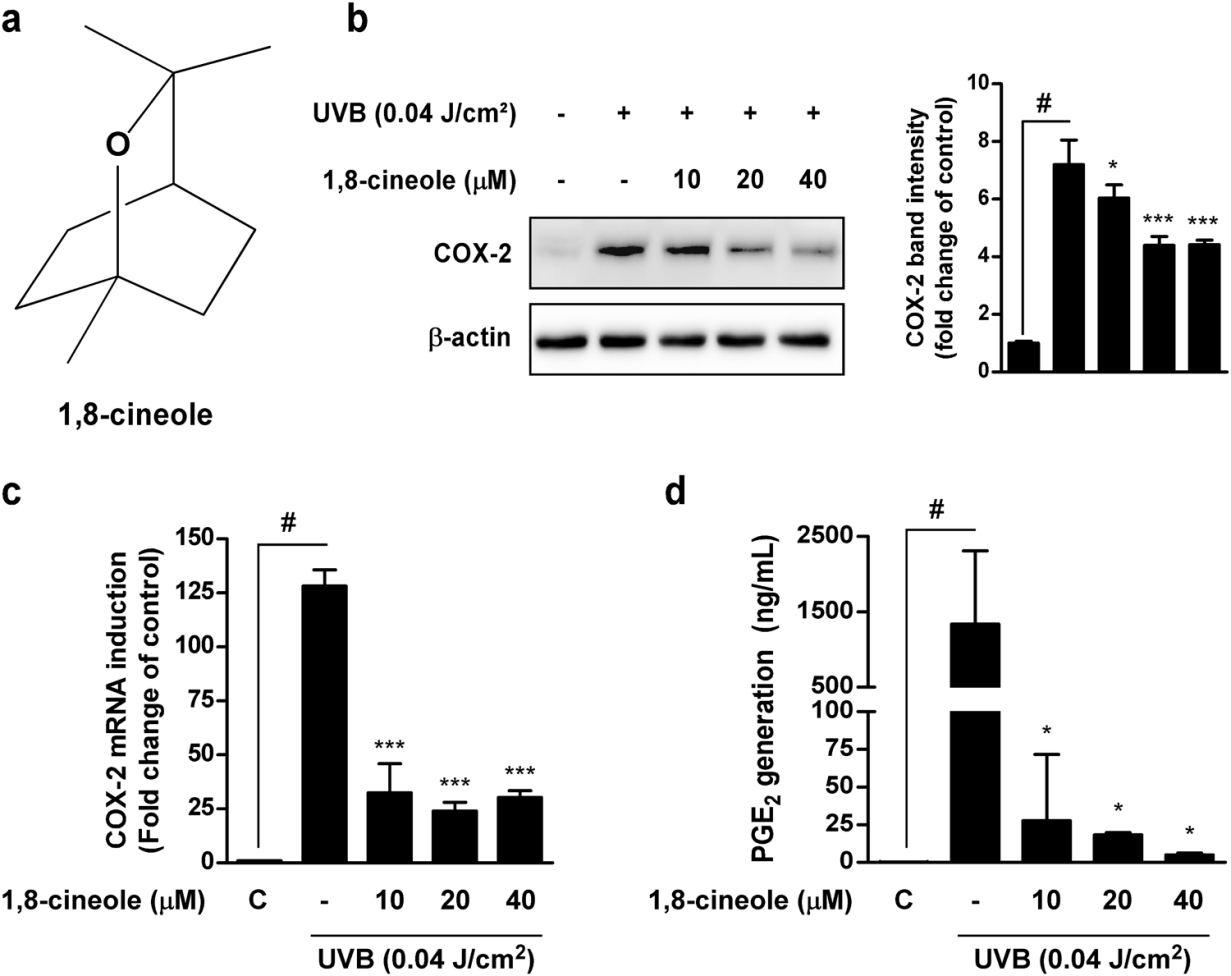
Effect of 1,8-cineole on UVB-induced COX-2 protein, mRNA expression, and PGE2 generation in HaCaT cells **(a)** Chemical structure of 1,8-cineole. **(b)** 1,8-cineole inhibits UVB-induced COX-2 protein expression in HaCaT cells. Cells were pre-treated with 1,8-cineole at the indicated concentrations for 1 hour, irradiated with UVB, and then harvested after 18 hours. Expression levels of COX-2 and β-actin were determined by Western blotting. Data are representative of three independent experiments that gave similar results. **(c)** 1,8-cineole inhibits UVB-induced COX-2 mRNA expression in HaCaT cells. COX-2 mRNA levels were measured by qRT-PCR. Cells were pre-treated with 1,8-cineole at the indicated concentrations for 1 hour, irradiated with UVB, and then total RNA was extracted after 4 hours. Data are represented as the mean ± SD of three independent experiments. **(d)** 1,8-cineole inhibits UVB-induced PGE_2_ generation in HaCaT cells. The quantity of PGE_2_ in the culture medium was measured by ELISA. Data are represented as the mean ± SD of three independent experiments. Hash symbol (^#^) indicates a significant difference between the control and the UVB-treated group; asterisk symbols (^*^ and ^***^) indicate significant differences (p < 0.05 and p < 0.001, respectively) between the groups treated with UVB and 1,8-cineole and the group treated with UVB alone.

Because abnormal expression of COX-2 is closely related to skin carcinogenesis, we first examined whether 1,8-cineole can inhibit UVB-induced COX-2 expression in HaCaT cells. 1,8-cineole significantly inhibited UVB-induced COX-2 protein (Figure 1b) and mRNA (Figure 1c) expression in HaCaT cells. 1,8-cineole also inhibited UVB-induced PGE_2_ generation in HaCaT cells (Figure 1d). Additionally, 1,8-cineole exhibited no cytotoxicity in HaCaT cells (Supplementary Figure 1).

### 1,8-cineole inhibits UVB-induced RAF-MEK1/2-ERK1/2 signaling in HaCaT cells

**Figure 2:**
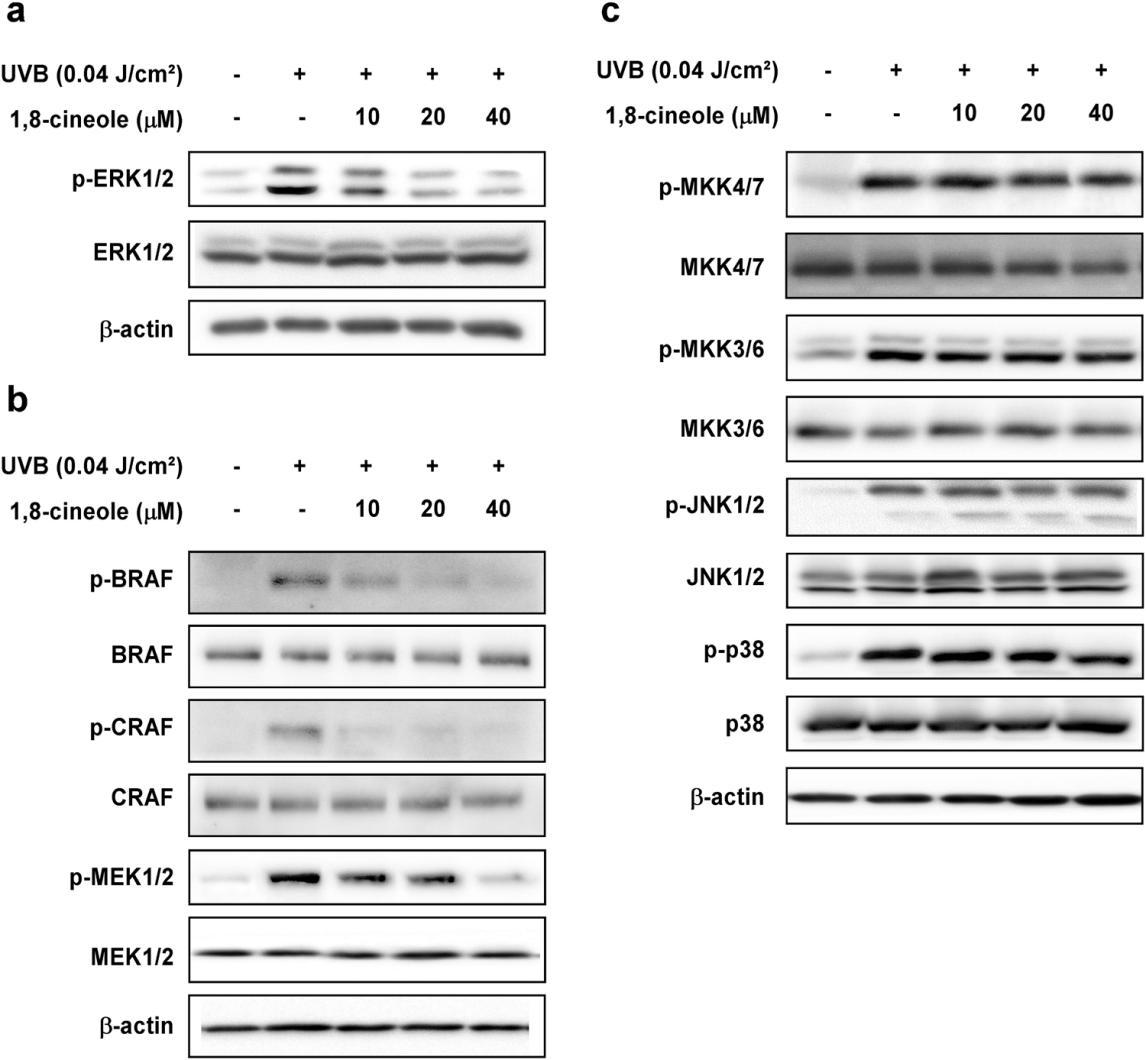
Effect of 1,8-cineole on UVB-induced phosphorylation of MAPKs in HaCaT cells **(a)** and **(b)** 1,8-cineole inhibits UVB-induced phosphorylation of ERK1/2, MEK1/2, BRAF, and CRAF, **(c)** but not MKK4/7, JNK1/2, MKK3/6, and p38 in HaCaT cells. Cells were pre-treated with 1,8-cineole at the indicated concentrations for 1 hour, irradiated with UVB, and then harvested after 15 min. Phosphorylation levels of proteins were detected by Western blotting.

**Figure 3:**
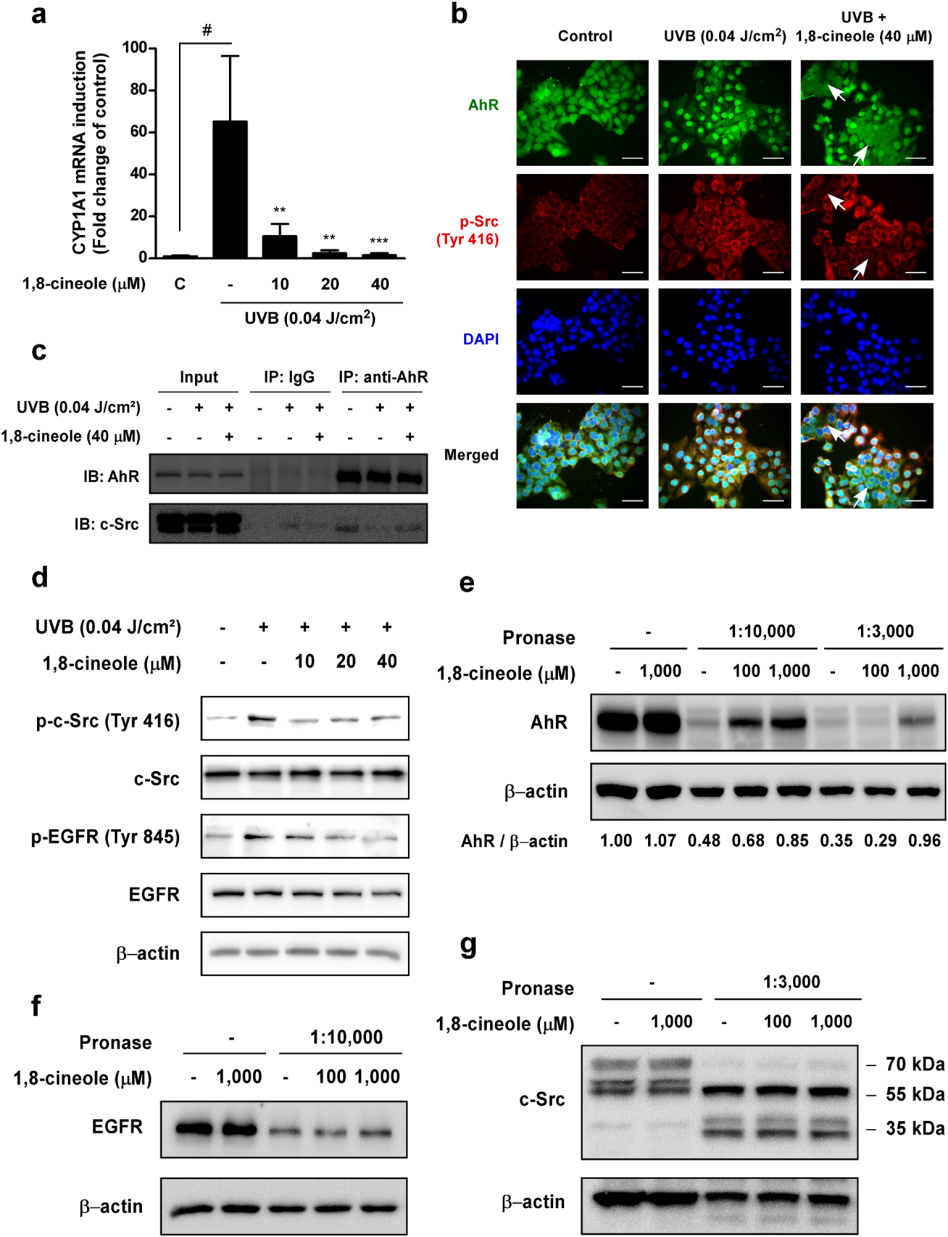
Effect of 1,8-cineole on UVB induction of the AhR/c-Src/EGFR signaling pathway and direct binding with AhR **(a)** 1,8-cineole inhibits UVB-induced phosphorylation of c-Src Tyr-416 and EGFR Tyr-845 residues in HaCaT cells. Cells were pre-treated with 1,8-cineole at the indicated concentrations for 1 hour, irradiated with UVB, and then harvested after 15 min. Phosphorylation levels of proteins were detected by Western blotting. **(b)** and **(c)** 1,8-cineole suppresses UVB-induced AhR nuclear translocation and dissociation from c-Src in HaCaT cells. Cells were pretreated with 1,8-cineole for 1 hours, before irradiation with UVB for 30 min. (b) AhR (green) and p-c-Src (Tyr 416) (red) were detected by fluorescence microscopy. Nuclei were counterstained with DAPI (blue). White arrows indicate inhibition of AhR nuclear translocation and c-Src phosphorylation by 1,8-cineole treatment in HaCaT cells. Scale bar, 50 μm. (c) Co-immunoprecipitation assay was performed asdescribedin the ‘Materials and Methods’ and levels of AhR and c-Src were detected by Western blotting. **(d)** 1,8-cineole inhibits UVB-induced CYP1A1 mRNA upregulation in HaCaT cells. CYP1A1 mRNA levels were measured by qRT-PCR. Cells were pre-treated with 1,8-cineole at the indicated concentrations for 1 hour, and irradiated with UVB before total RNA was extracted after 4 hours. **(e)** 1,8-cineole directly binds to AhR but not **(f)** EGFR or **(g)** c-Src in HaCaT cells. For the DARTS assay, cells were pre-treated with 1,8-cineole at the indicated concentrations for 1 hour and lysed. The lysates were digested with pronase (at the indicated pronase to protein ratio) and subjected to Western blotting.

Previous studies have shown that activation (Figure 3g) proteolysis suggesting that 1,8- cineole does of the MAPK signaling pathway by UVB irradiation upregulates *cox-2* gene expression [7]. Thus, we further examined whether 1,8-cineole affects UVB-induced activation of the MAPK and MAPKK pathways. Of the MAPK family members examined, 1,8-cineole only inhibited UVB-induced phosphorylation of ERK1/2 (Figure 2a), and phosphorylation of its upstream regulators MEK1/2, BRAF and CRAF (Figure 2b), while not affecting UVB-induced phosphorylation of MKK4/7- JNK1/2 or MKK3/6 - p38 MAPK signaling axis (Figure 2c).

### 1,8-cineole inhibits UVB-induced AhR/c-Src/EGFR signaling in HaCaT cells

UVB-induced AhR activation upregulates suppression of reversible tumor promotion before tumor the RAF-MEK1/2-ERK1/2 signaling pathway via c-Src-dependent EGFR activation, and when AhR is activated, it upregulates its specific target gene, *cyp1a1* [12]. We next examined whether 1,8-cineole could affect UVB-induced *cyp1a1* mRNA expression. 1,8-cineole significantly inhibited UVB-induced *cyp1a1* mRNA upregulation in a dose-dependent manner (Figure 3a). We then investigated whether 1,8-cineole downregulates the AhR/c-Src/EGFR signaling pathway. Immunofluorescence staining and co-immunoprecipitation results showed that 1,8-cineole suppresses UVB-induced AhR nuclear translocation and dissociation from c-Src (Figure 3b and 3c). Western blot results showed that 1,8-cineole inhibited UVB-induced phosphorylation of c-Src and EGFR residues (Figure 3d). Because UVB-induced intracellular ROS generation can also upregulate COX-2 expression via the phosphorylation of Akt and EGFR [21, 22], we examined whether 1,8-cineole may affect ROS-dependent COX-2 expression. 1,8-cineole did not suppress UVB-induced intracellular ROS generation (Supplementary Figure 2a and 2b) or phosphorylation of its downstream regulators, Akt and EGFR (Supplementary Figure 2c).

### 1,8-cineole binds directly to AhR in HaCaT cells

Because 1,8-cineole suppressed UVB-induced transactivation of AhR and activation of its specific downstream regulators, we hypothesized that 1,8-cineole may specifically inhibit UVB-induced AhR activity via direct binding to AhR. To confirm the molecular target of 1,8-cineole, we conducted a drug affinity responsive target stability (DARTS) assay using HaCaT cells. DARTS analysis revealed that 1,8-cineole directly binds to AhR and inhibits its proteolysis (Figure 3e). Because direct targeting of c-Src and EGFR can also regulate the RAF-MEK1/2-ERK1/2 signaling pathway, we investigated whether the binding of 1,8-cineole to AhR was specific. 1,8- cineole failed to block EGFR (Figure 3f) and c-Src (Figure 3g) proteolysis suggesting that 1,8- cineole does not bind to these proteins.

### Knockdown of AhR inhibits UVB-induced COX-2 expression and phosphorylation of ERK1/2 in HaCaT cells

**Figure 4:**
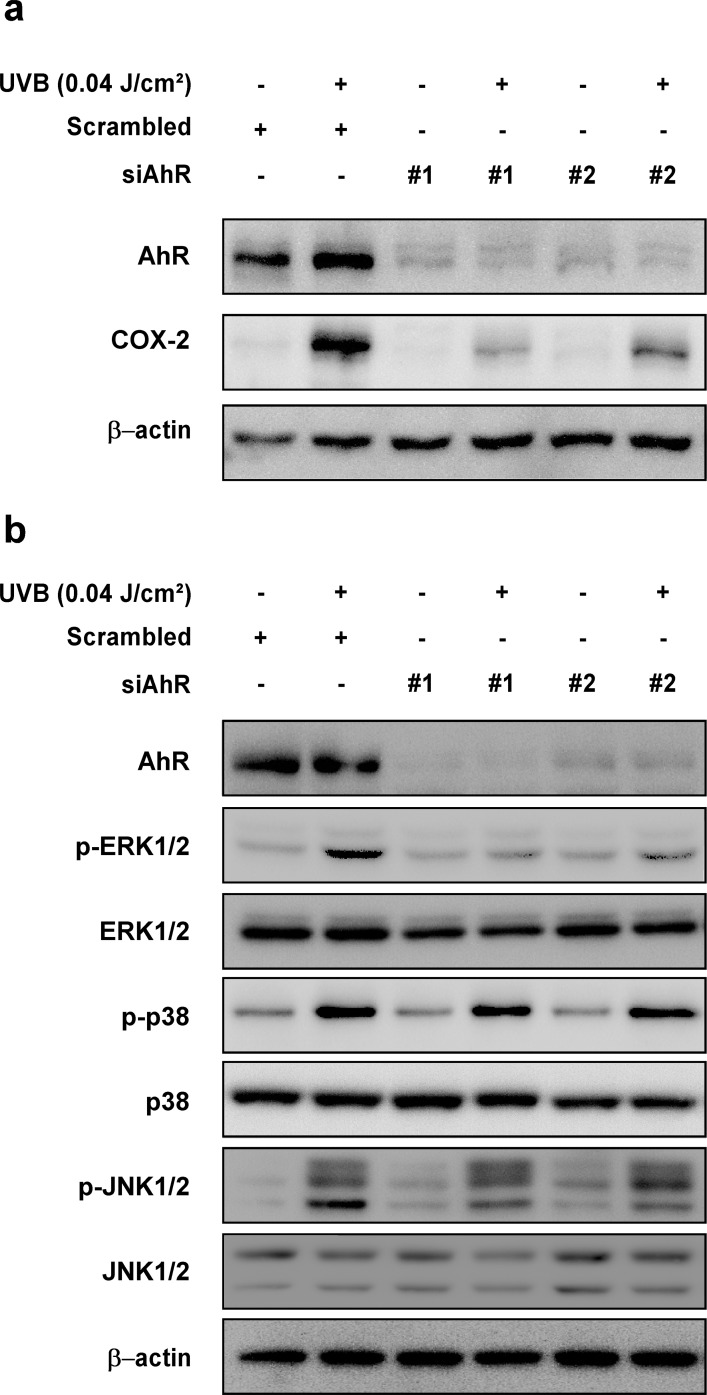
Effect of AhR knockdown on UVB-induced COX-2 expression and phosphorylation of ERK1/2, p38, and JNK1/2 in HaCaT cells **(a)** Knockdown of AhR reduced UVB-induced COX-2 expression in HaCaT cells. Cells were transfected with scrambled (negative control) or indicated siRNA for 24 hours, irradiated to UVB for 18 hours. Expression levels of proteins were detected by Western blotting. **(b)** Knockdown of AhR reduced UVB-induced phosphorylation of ERK1/2, but not p38 and JNK1/2. Cells were transfected with scrambled (negative control) or indicated siRNA for 24 hours, irradiated to UVB for 15 min. Phosphorylation levels of the proteins were detected by Western blotting.

To further confirm the role of AhR on UVB-induced COX-2 expression and activation of upstream MAPK signaling, we transfected HaCaT cells with siRNA for AhR knockdown. Knockdown of *AhR* reduced AhR protein levels and suppressed UVB-induced COX-2 protein expression (Figure 4a) and phosphorylation of ERK1/2, but not p38 or JNK1/2 (Figure 4b).

### 1,8-cineole suppresses UVB-induced skin tumorigenesis *in vivo*

**Figure 5:**
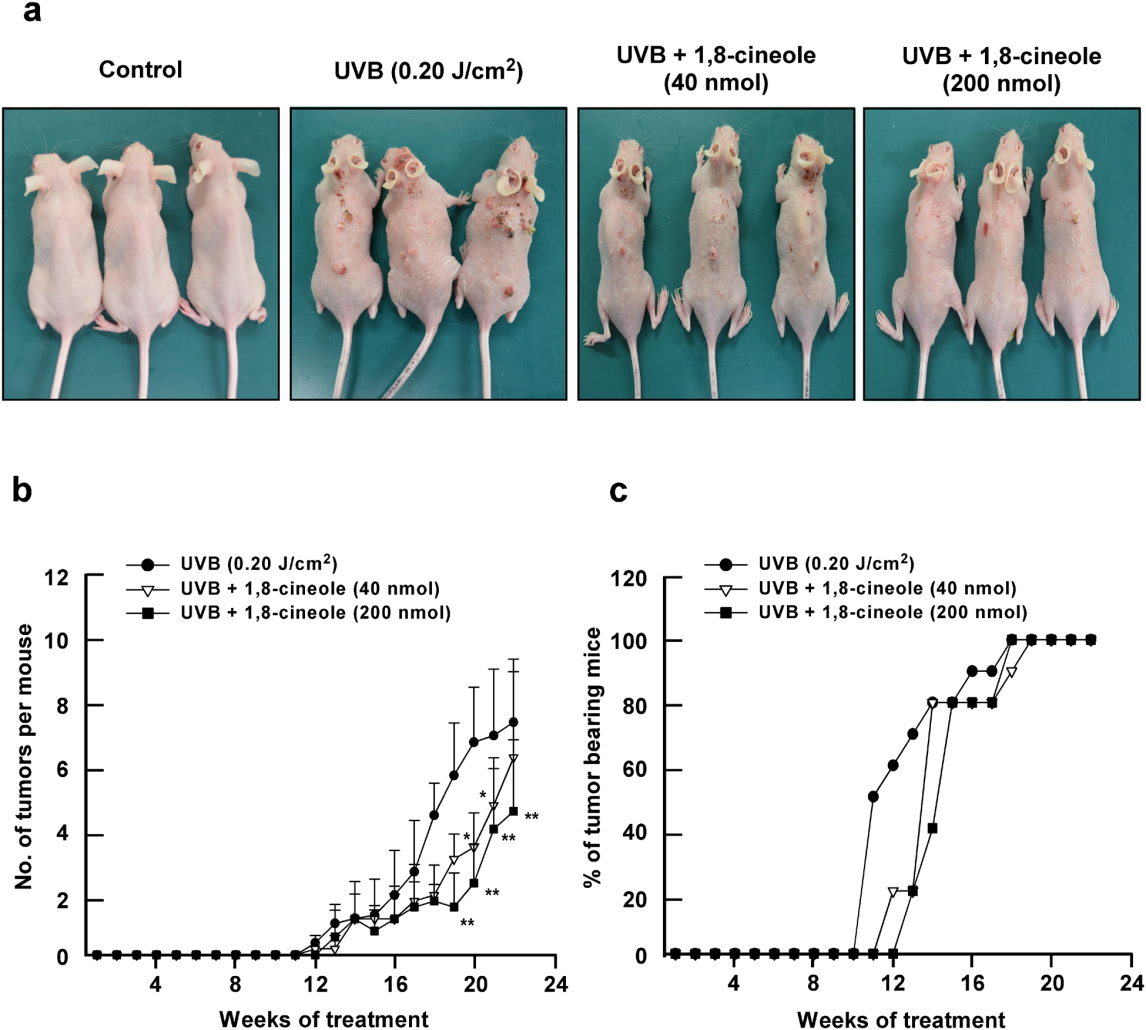
Effect of 1,8-cineole on UVB-induced skin tumorigenesis in SKH-1 hairless mouse skin **(a)** The external appearance of mice at 22 weeks after UVB-treatment. The mice in control group (n = 5) were subjected to the topical treatment of 200 μL acetone on dorsal skin without UVB irradiation (0.20 J/cm^2^) at 3 days/week for 22 weeks. The mice in UVB-irradiated groups (n = 5 per each group) were treated with 200 μL acetone or the indicated amount of 1,8-cineole (40 nmol or 200 nmol) in 200 μL acetone topically on the dorsal skin for 1 hour before UVB irradiation (0.20 J/cm^2^) for 3 days/week for 22 weeks. **(b)** 1,8-cineole suppresses UVB-induced tumor incidence in SKH-1 hairless mice. The incidence of skin tumors was recorded weekly. A tumor was defined as an outgrowth >1 mm in diameter that persisted for 2 weeks or longer. Tumor incidence and multiplicity were recorded each week until the end of the experiment at 22 weeks. Asterisk symbols (^*^ and ^**^) indicates a significant difference (p < 0.05 and p < 0.01, respectively) between the UVB-treated and the UVB + 1,8-cineole-treated groups. **(c)** 1,8-cineole reduces the number of UVB-induced tumour–bearing mice. A tumor was defined as described above and the number of tumor-bearing mice was measured weekly for 22 weeks.

To investigate the preventive effect of 1,8-cineole on UVB-induced skin carcinogenesis *in vivo*, we used a two-stage skin carcinogenesis model with SKH-1 hairless mice. Topical treatment of 1,8-cineole on the dorsal skin of SKH-1 mice significantly suppressed skin tumorigenesis after 22 weeks of chronic UVB-treatment (Figure 5a). 1,8-cineole treatment (40 or 200 nmol) reduced the number of tumors by 38.2% and 48.4% compared to the UVB-only irradiated group, respectively. Although skin tumors developed after 10 weeks in the UVB-only-treated group, low and high dose treatment of 1,8-cineole delayed development of these tumors by 1 or 2 weeks, respectively, in SKH-1 mice (Figure 5c). These findings suggest that 1,8-cineole inhibits UVB-induced skin carcinogenesis *in vivo*.

### 1,8-cineole suppresses UVB-induced COX-2 expression and skin hyperplasia *in vivo*

**Figure 6:**
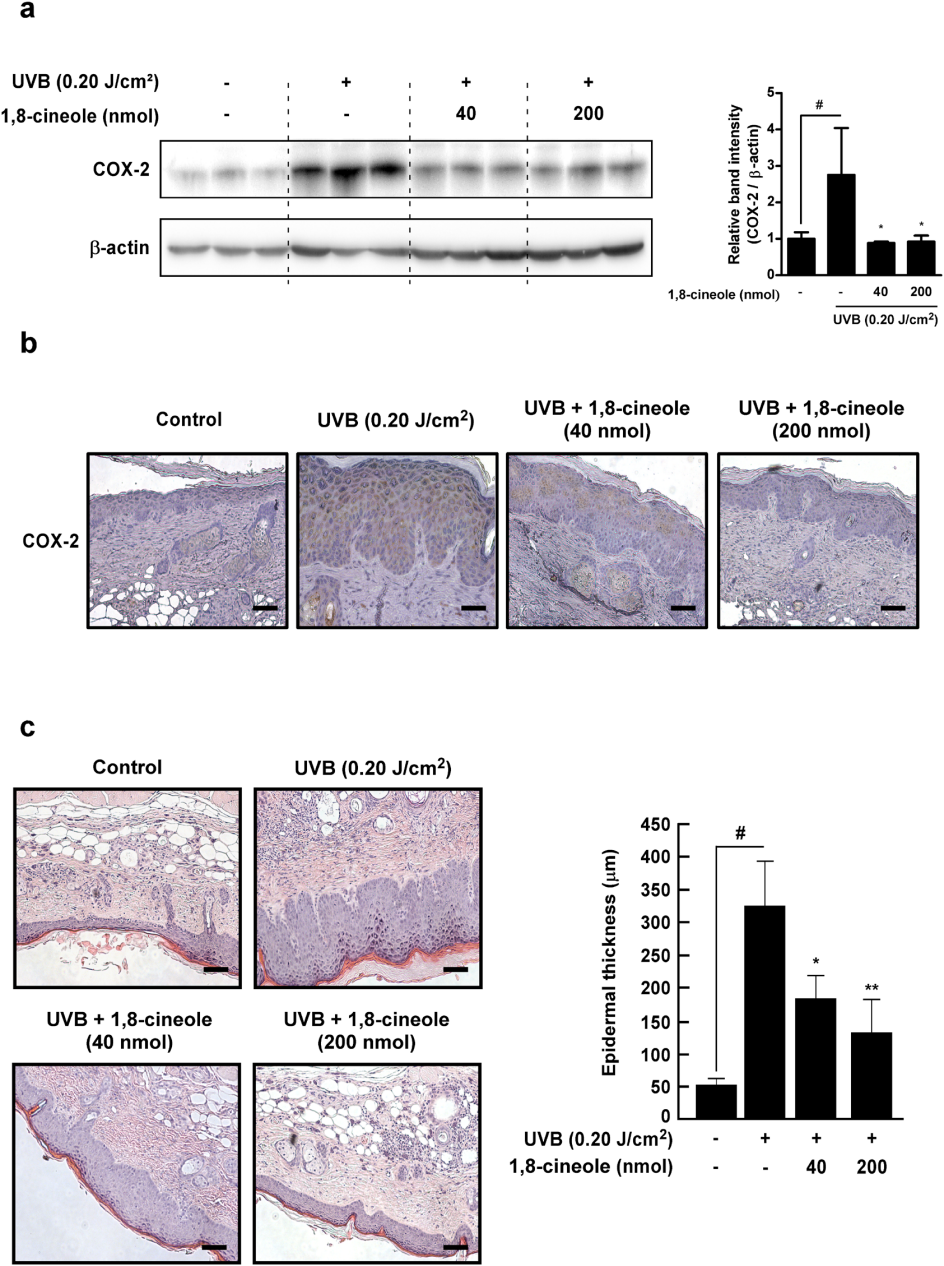
Effect of 1,8-cineole on UVB-induced COX-2 expression and epidermal hyperplasia in SKH-1 hairless mouse skin **(a)** and **(b)** 1,8-cineole inhibits UVB-induced COX-2 expression in SKH-1 mouse skin. Expression levels of proteins were detected by Western blotting. Each band was densitometrically quantified by image analysis. The band density of COX-2 was normalized to β-actin followed by statistical analysis. Quantification data are represented as means ± SD (n = 3). Immunohistochemical analysis shows representative photographs of overall staining patterns from each group. The expression level of COX-2 proteins was stained as brown and the nucleus was counterstained as blue. Scale bar, 20 μm. **(c)** 1,8-cineole inhibis UVB-induced epidermal hyperplasia in SKH-1 mouse skin. Hematoxylin- and eosin-staining shows representative photographs of overall staining patterns from each group. Scale bar, 20 μm. Bars graph represent epidermal thickness (μm) of the indicated groups. Hash symbol (^#^) indicates a significant difference between the control and the UVB-treated group (p < 0.05); asterisk symbols (^*^ and ^**^) indicate significant differences (p < 0.05 and p < 0.01) between the groups treated with UVB and 1,8-cineole and the group treated with UVB alone.

We next examinedwhether 1,8-cineole regulates UVB-induced COX-2 expression in SKH-1 hairless mice. Western blot results showed that 1,8-cineole suppresses UVB-induced COX-2 expression in the skin (Figure 6a). Immunohistochemistry results also showed that 1,8-cineole inhibits UVB-induced COX-2 expression in the SKH-1 mice (Figure 6b). Epidermal COX-2 expression induced by UVB irradiation dysregulates keratinocyte function and contributes to epidermal hyperplasia, leading to epithermal thickening, a marker of skin damage [23]. 1,8-cineole treatment significantly reduced UVB-induced thickening of the epidermis in a dose-dependent manner in the SKH-1 hairless mice (Figure 6c). These results suggest that treatment with 1,8-cineole inhibits UVB-induced epidermal COX-2 expression and hyperplasia *in vivo*.

## DISCUSSION

Chemoprevention may represent a more effective strategy for the management of cancer rather than therapeutic approaches because it suppresses tumor promotion prior to the formation of malignant lesions [7]. The World Health Organization (WHO) indicates that approximately 35% of all deaths caused by cancer are preventable, and some phytochemicals have been reported to prevent carcinogenesis by inhibiting the cellular signaling pathways involved in tumor promotion [14]. We have previously demonstrated that some compounds derived from natural products have a preventive effect against UVB-induced skin carcinogenesis by inhibiting the MAPK signaling pathway [24, 25]. Chronic skin inflammation is one of the hallmark effects of UV-irradiation and plays a critical role in the development of skin cancer [4, 5], for which COX-2 is known to be a critical regulator [26, 27]. We have observed that *Curcuma zedoaria* extract, an abundant source of 1,8-cineole, inhibits UVB-induced skin inflammation by regulating COX-2 expression and the upstream MAPK signaling pathway (unpublished data). However, the preventive effect of 1,8-cineole on skin carcinogenesis and the molecular mechanisms of action responsible have remained unknown. In the present study, we sought to examine the mechanisms of action responsible for the effect of 1,8-cineole on UVB-induced skin tumorigenesis in HaCaT human keratinocytes and SKH-1 hairless mice.

The AhR signaling pathway is one of the major UVB irradiation-induced signaling pathways that regulate COX-2 expression [6, 12]. COX-2 expression is up-regulated following the activation of AhR, which in turn leads to c-Src-mediated EGFR activation and subsequent ERK activation [12]. Interestingly, we observed that 1,8-cineole only suppressed UVB-induced ERK phosphorylation, but not p38 or JNK phosphorylation. Upon AhR activation, c-Src is activated by phosphorylation of its Tyr 416 residue before the activated c-Src phosphorylates Tyr 845 on EGFR for its activation [28]. Our Western blot analysis showed that 1,8-cineole inhibits UVB-induced phosphorylation of c-Src at Tyr 416 and EGFR at Tyr 845. In addition, immunofluorescence staining showed that 1,8-cineole inhibited UVB-induced phosphorylation of c-Src at Tyr416 and dissociation from AhR. Findings from a previous study suggest that phosphorylation of c-Src at Tyr 416 is closely associated with the phosphorylation of EGFR at Tyr 845. Therefore, inhibiting c-Src phosphorylation and the dissociation of c-Src from AhR due to 1,8-cineole treatment may affect EGFR activity and subsequently suppress COX-2 expression. ROS generated by UVB irradiation can induce the phosphorylation of EGFR at Tyr 1068 and Akt at Ser 473 to upregulate COX-2 expression [21, 22], but 1,8-cineole did not affect phosphorylation of these targets. These findings suggest that 1,8-cineole specifically inhibits UVB-induced activation of the AhR/c-Src/EGFR signaling pathway and subsequent COX-2 expression.

Activator protein-1 (AP-1) and cAMP response element-binding protein (CREB) were known to be susceptible to UVB-induced COX-2 expression via MAPK signaling pathways [7, 29, 30]. However, we confirmed that 1,8-cineole does not have a significant impact on UVB-induced transactivation of AP-1 (Supplementary Figure 3a) or phosphorylation of CREB at Ser 133 (Supplementary Figure 3b) in HaCaT cells. To further investigate which transcription factors are primarily regulated by 1,8-cineole treatment, we conducted a transcriptome analysis using an mRNA microarray in HaCaT cells (UVB-only treated vs. UVB + 1,8-cineole-treated group). A bioinformatics tool, TFactS [31] predicted that NF- κB (*p* = 0.00028) and STAT1 (*p* = 0.0016) were transcription factors that could be regulated by 1,8-cineole in HaCaT cells (Data not shown). However, further investigation is needed to determine the transcription factors involved in UVB-induced COX-2 expression via the AhR/c-Src/EGFR/ERK pathway.

Molecular target identification of small molecules that modulate the function of target proteins is important to understand their effect on disease progression [32]. Recently, a number of cancer preventive agents have been developed based on the identification of precise molecular targets. For example, the FDA-approved drug tamoxifen directly targets the estrogen receptor and elicits a preventive effect against breast cancer [33]. Affinity-based methods are currently used to identify the protein targets of small molecules, but are often subject to chemical modification and immobilization [34]. Although the pull-down assay, a core affinity-based target identification method, has identified the molecular targets of phytochemicals that inhibit UVB-induced COX-2 expression [24, 25, 35, 36], identification has been limited to polyphenolic compounds because this method requires multiple reactive hydroxyl groups to ensure proper immobilization. In addition, this method can conceivably modify the chemical moieties that interact with targets [37]. In this study, we first observed that the natural monoterpenoid 1,8-cineole, which does not contain hydroxyl moieties, exhibits inhibitory effects against UVB-induced COX-2 expression by directly targeting AhR. DARTS is a method that can be used to identify the molecular targets of small molecules without chemical modification, and has been used to successfully identify the molecular targets of some anti-cancer drugs [38, 39] and phytochemicals [40, 41] without structural limits. However, DARTS has some limitations including drug binding affinity to its target as a limiting factor, the possibility of modifying protease susceptibility of non-target proteins following drug treatment, and low sensitivity with mass spectrometry [32]. We also confirmed that knockdown of AhR reduced UVB-induced COX-2 expression via the inhibition of ERK phosphorylation. Consistent with our results, previous studies have reported that pharmacological inhibition of AhR is effective in suppressing UVB-induced COX-2 expression [12, 36]. These results suggest that 1,8-cineole directly binds to AhR and suppresses UVB-induced COX-2 expression via the ERK signaling pathway. For most cases in which the DARTS assay is used, small molecules are directly added to the cell lysates [42], but we observed that 1,8-cineole binds to AhR when pretreated to cells, but not when added to cell lysates (data not shown). Based on these observations, we hypothesized that 1,8-cineole is metabolized inside the cell and then binds to AhR. However, identification of 1,8-cineole metabolites that bind to AhR and the mechanism of action responsible for regulating AhR activity has not been previously investigated.

Upregulated COX-2 expression followed UVB irradiation is a common hallmark of skin carcinogenesis [26, 27]. In the present study, 1,8-cineole delayed and reduced the incidence of tumors and suppressed UVB-induced COX-2 expression in SKH-1 hairless mice. A previous study has demonstrated that COX-2 expression in skin epidermal cells plays a pivotal role in UVB-induced skin carcinogenesis, while c*ox-2* gene deletion reduces UVB-induced epidermal hyperplasia and skin tumor incidence in SKH-1 hairless mice [43]. Consistent with the previous study, our results showed that 1,8-cineole reduces UVB-induced epidermal thickening in the SKH-1 mice. These results suggest that 1,8-cineole suppresses UVB-induced tumor promotion and epidermal thickening by inhibiting COX-2 expression *in vivo*.

Taken together, our findings shed light on the chemopreventive effect of 1,8-cineole on skin cancer induced by UVB irradiation and the mechanism of action responsible. 1,8-cineole may have applications as a natural ingredient for the prevention of UVB-induced skin carcinogenesis. Other environmental toxins, such as TCDD, particulate matters, and benzo[*a*]pyrene, have been reported to promote metabolic diseases through AhR activation [8]. Further studies are therefore warranted to investigate the potential effect of 1,8-cineole on other AhR-mediated diseases.

## MATERIALS AND METHODS

### Reagents and antibodies

1,8-cineole (99%) and *N*-Acetyl-L-cysteine (NAC, 99%) were purchased from Sigma Aldrich (St. Louis, MO, USA). Antibodies specific to detect Ser217/221-phospho MEK, total MEK, Ser257/Thr261-phospho MKK4/7, total MKK4/7, Ser178/207-phospho MKK3/6, total MKK3/6, Thr202/Tyr204-phospho ERK, total ERK, Tyr180/182-phospho p38, total p38, Thr183/Tyr185-phospho JNK, total JNK, Ser455-phospho BRAF, Ser338-phospho CRAF, Tyr416-phospho Src, Tyr845 and Tyr1068-phospho EGFRs, total EGFR, Ser473-phospho Akt, total Akt, COX-2, Ser133-phospho CREB, and CREB were obtained from Cell Signaling Biotechnology (Beverly, MA, USA). Antibodies specific to total BRAF (F-7), total CRAF (C-12), AhR (H-211), c-Src (B-12), and β-actin (C-4) were obtained from Santa Cruz Biotechnology (Santa Cruz, CA, USA). The protein assay kit was obtained from Bio-Rad Laboratories (Hercules, CA, USA).

### Cell culture and viability assay

Human epidermal keratinocyte HaCaT cells were maintained in DMEM containing 10% FBS (Gibco, Grand Island, NY, USA), 100 U/ml of penicillin and 100 mg/ml of streptomycin at 37°C in a 5% CO_2_ humidified incubator. The UVB light source (Bio-Link Crosslinker; Vilber Lourmat, Marne-la-Vallée, France) emitted wavelengths of 254, 312, and 365 nm, with peak emission at 312 nm. To estimate cell viability, HaCaT cells were seeded (10^3^ class=apple-converted-space> cells/well) in 96-well plates and incubated at 37°C in a 5% CO_2_ class=apple-converted-space> incubator. After the cells were treated with 1,8-cineole, 100 μl of MTS solution in the presence of phenazine methosulphate was added to each well. After 1 hr of incubation, the absorbance levels for formazan at 490 and 690 nm were measured by using a microplate reader.

### Western blot analysis

For Western blot assay, cells (1.5 × 10^6^ total) were cultured in a 10-cm dish for 48 hrs, followed by starvation in serum-free DMEM for 24 hrs. Cells were then treated with 1,8-cineole (10, 20, or 40 μM) for 1 hr and irradiated with UVB (0.04 J/cm^2^). The protein concentration was determined by using a dye-binding protein assay kit (Bio-Rad Laboratories) following instructions in the manufacturer's manual. Lysate protein was subjected to 10% SDS-PAGE and transferred to a polyvinylidene difluoride membrane (Millipore, Billerica, MA, USA). After transferring, the membranes were incubated with specific primary antibodies at 4°C overnight. Protein bands were visualized by using a chemiluminescence detection kit (Thermo Scientific, Waltham, MA, USA) after hybridization with a horseradish peroxidase-conjugated secondary antibody.

### PGE_2_ ELISA assay

1,8-cineole was treated to HaCaT cells plated in 6-well dishes at 80% confluency, 1 hr prior to UVB (0.04 J/cm^2^) irradiation, and then harvested 18 hours later. The quantity of PGE2 released into the medium was measured by using a PGE2 enzyme immunoassay kit (Enzo Life Science, Farmingdale, NY, USA).

### Quantitative real-time RT-PCR (qRT-PCR)

Total RNA was isolated using the RNeasy^®^ Mini Kit (Qiagen, Valencia, CA, USA) according to the manufacturer's instructions. Reverse transcription of RNA was performed with the ReverTra Ace^®^ qPCR RT Master Mix (Toyobo, Osaka, Japan). First-strand cDNA was prepared from 1 μg total RNA. The real-time PCR reaction was performed in a volume of 20 μl containing 0.1 μg of cDNA, 1 μM of each primer (human COX-2 sense 5′-CCC TTG GGT GTC AAA GGT AA-3′ and antisense 5′-AAC TGA TGC GTG AAG TGC TG-3′, human CYP1A1 sense 5′-TAG ACA CTG ATC TGG CTG CAG-3′ and antisense 5′-GGG AAG GCT CCA TCA GCA TC), and Power SYBR^®^ Green PCR Master Mix (Applied Biosystems, Carlsbad, CA). The thermal cycling was carried out in a StepOnePlus Real-Time PCR system (Applied Biosystems) with a program of 95°C for 5 min., followed by 40 cycles with denaturation at 95°C for 5 sec., annealing and elongation at 60°C for 10 sec. The gene expression levels were normalized to the expression level of the GAPDH housekeeping gene (human GAPDH anti sense 5′-GAA GGT GAA GGT CGG AGT-3′ and antisense 5′-CAT GGG TGG AAT CAT ATT GGA A-3′. Relative gene expression changes, calculated using the 2^−ΔΔCT^ method, are reported as number-fold changes compared to those in the control samples.

### Drug affinity responsive target stability (DARTS) assay

The DARTS assay was performed according to the protocol previously described [32]. HaCaT cells were treated with 1,8-cineole or DMSO plated in 10-cm dishes at 80% confluency, before the cells were lysed after 1 hour in M-PER buffer (Pierce, Rockford, IL, USA) containing protease and phosphatase inhibitors. After centrifugation (12,000 rpm, 10 min, 4°C), 10x TNC buffer [500mM Tris·HCl (pH 8.0), 500mM NaCl, 100 mM CaCl_2_] was added to the lysates, before the protein concentration was determined with a dye-binding protein assay kit (Bio-Rad Laboratories) following the manufacturer's instructions. The lysates (2.5 μg/ μL) were digested with pronase (1: 3,000 or 1: 10,000 of protein to pronase ratio) for 30 min. Digestion was stopped by adding 5x Laemmli sample buffer, before the samples were subjected to Western blot analysis.

### Immunofluorescence

HaCaT cells were seeded (8 × 10^3^ cells/well) in 96-well plates and incubated at 37°C for 24 hours in a 5% CO_2_ incubator. The medium was changed with 1,8-cineole for 1 hr. The cells were then exposed to UVB irradiation (0.04 J/cm^2^) and incubated for 30 min. Cells were fixed with 4% paraformaldehyde, permeabilized with 0.3% Triton X-100 and stained with anti-rabbit AhR and anti-mouse c-Src antibodies, before visualization with goat anti-rabbit IgG-h+l conjugated to DyLight® 488 conjugated labeled secondary antibodies and goat anti-mouse IgG-h+l conjugated to DyLight® 594-conjugated labeled secondary antibodies (Bethyl Laboratories, TX, USA). The nuclei were counterstained with DAPI (Thermo Scientific, Waltham, MA, USA) and the cells were visualized using fluorescence microscopy (Nikon Eclipse Ti-S, Tokyo, Japan). Images were analyzed using Metamorph (Molecular Devices, Danville, PA) software.

### Co-immunoprecipitation assay

HaCaT cells were seeded in 10-cm dishes (1 × 10^6^ cells/dish) and incubated at 37°C for 24 hours in a 5% CO_2_ incubator. The cells were then starved with serum-free medium for 24 hours, before the medium was changed with 1,8-cineole for 1 hr. The cells were then exposed to UVB irradiation (0.04 J/cm^2^) and incubated for 30 min. The HaCaT cells were lysed in M-PER lysis buffer (Pierce, Rockford, IL, USA) containing protease and phosphatase inhibitors. The cell lysate containing 500 mg of protein was incubated with anti-IgG (Cell Signaling Biotechnology; Beverly, MA, USA) or anti-AhR (Santa Cruz, CA, USA) overnight at 4°C. Then, 20 μL of a 50% slurry of protein G agarose beads (Pierce, Rockford, IL, USA) was added to the samples followed by incubation for 2 hours at 4°C. The samples were centrifuged for 1 min at 14,000 g and washed with lysis buffer five times. The pellets were resuspended with 20 uL of 3x SDS sample buffer and the samples were heated at 95°C for 5 min for subsequent Western blotting assay.

### Knockdown of AhR

For knockdown of AhR, HaCaT cells were transfected with scrambled (Cat No. SN-1002, Bioneer, Daejeon, Korea) or 10 μM human AhR siRNA (Cat No. 1003685 (#1), 1003681 (#2), Bioneer) by using Lipofectamine^®^ RNAiMAX (Invitrogen, Carlsbad, CA, USA), following the manufacturer's suggested protocols. The transfected cells were then exposed to UVB irradiation and used in subsequent experiments.

### Animal experiments

SKH-1 hairless mice (6 weeks of age; mean body weight, 25 g) were purchased from Central Lab Animal Inc. (Seoul, Korea). Animals were acclimated for 1 week before the study and had free access to food and water. The animals were housed in climate-controlled quarters (24°C at 50% humidity) with a 12-h light/12-h dark cycle. All animals received humane care, and the study protocol (KFRI-M-14013) was approved and performed in accordance with the guidelines for animal use and care at Korea Food Research Institute. Skin carcinogenesis was induced using a UVB irradiation system in mice. The UVB radiation source (Bio-Link cross-linker; Vilber Lourmat) emitted at wavelengths of 254, 312, and 365 nm, with peak emission at 312 nm. SKH-1 mice were divided into four groups of 5 animals each. In the control mice, the dorsal skin was topically treated with 200 μL acetone only. In the UVB group of mice, the dorsal skin was topically treated with 200 μL acetone 1 h before UVB. The mice in the third and fourth groups received topical application of 1,8-cineole (40 or 200 nmol) in 200 μL acetone 1 h before UVB irradiation. The UVB dose was 0.18 J/cm^2^ given thrice/wk for 22 wk. The incidence of skin tumors was recorded weekly, and a tumor was defined as an outgrowth of >1 mm in diameter that persisted for 2 weeks or more. Tumor incidence, multiplicity, and volume were recorded every week until the end of the experiment at the 22th week.

### Immunohistochemical analysis

Dorsal skin from the mice was prepared for immunohistochemical analysis of COX-2 expression. Sections (5 μm thick) of 10% neutral formalin solution- fixed paraffin-embedded tissues were cut on silane-coated glass slides and then deparaffinized three times with xylene and dehydrated through a graded alcohol bath. The deparaffinized sections were incubated in 20 μg/ml proteinase K for 20 min at room temperature for antigen retrieval. To prevent non-specific staining, each section was treated with 3% hydrogen peroxide for 20 min and a blocking solution containing 1% bovine serum albumin for 2 h. For the detection of the target protein, the slides were incubated overnight with an affinity-purified primary antibody at 4°C in 1% bovine serum albumin and then developed using an anti-rabbit or anti-mouse Histostain Plus Kit (Zymed Laboratories, South San Francisco, CA). Peroxidase-binding sites were detected by staining with 3,3′-diaminobenzidine tetrahydrochloride (Sigma–Aldrich). Mayer's hematoxylin was applied as a counterstain (Sigma–Aldrich).

### Measurement of epidermal thickness

Dorsal skin from mice was embedded in 10% formaldehyde and cut into 6-μm-thick sections under a microscope (Cryostat CM3050S, Leica Biosystems, St. Gallen, Switzerland). The sections were stained with hematoxylin and 0.5% eosin (Sigma; hematoxylin and eosin (H & E) staining) to measure the epidermal thickness of the ear tissue samples. Thickness was analyzed using Micrometrics SE Premium software (ACCU-SCOPE, Commack, NY, USA).

### Measurement of intracellular ROS

HaCaT cells (8 × 10^3^ cells/well) were seeded in 96-well plates and incubated at 37°C in a 5% CO_2_ incubator. When cells reached 80–90% confluence, they were starved by culturing in serum-free DMEM for a further 24 hrs. Cells were treated with 1,8-cineole (10, 20, or 40 μM) for 1 hr before treatment with 5 μM H_2_DCFDA (2,7-dichlorodihydrofluorescein diacetate, Molecular Probes, Eugene, OR, USA) for 30 min prior to UVB (0.04 J/cm^2^) exposure. Intracellular ROS generation was measured immediately following UVB exposure with a fluorometer (SpectraMax M2, Molecular Devices, Bath, UK) at 485/530 nm and visualized using fluorescence microscopy (Nikon Eclipse Ti-S, Tokyo, Japan).

### Luciferase assay for AP-1 transactivation

Confluent monolayers of HaCaT cells stably transfected with an AP-1 luciferase plasmid [44] were harvested, and 8 × 10^3^ viable cells suspended in 100 μL of 10% FBS/DMEM were added to each well of a 96-well plate. Plates were incubated at 37°C in 5% CO2. When cells reached 80–90% confluence, they were starved by culturing in 0.1% FBS–DMEM for another 24 hrs. The cells were then treated for 1 hr with 1,8-cineole before exposure to UVB (0.04 J/cm^2^), and then incubated for an additional 4 hrs. Cells were disrupted with 100 μL lysis buffer [24] and luciferase activity was measured using a luminometer (SpectraMax L; Molecular Devices, Sunnyvale, CA, USA).

### Statistical analysis

Where appropriate, data are expressed as the mean ± SD or SEM, and significant differences were determined by using one-way ANOVA. A probability value of P < 0.05 was used as the criterion for statistical significance.

## SUPPLEMENTARY MATERIALS FIGURES


