# Supplementary material for: 1,8-cineole prevents UVB-induced skin carcinogenesis by targeting the aryl hydrocarbon receptor
Source: Oncotarget. 2017 Nov 20;8(62):105995–6008. doi: 10.18632/oncotarget.22519 (PMC5739696; doi:10.18632/oncotarget.22519)
Supplement: Supplementary file 1 [file oncotarget-08-105995-s001.pdf]

1,8-cineole prevents UVB-induced skin carcinogenesis by targeting the aryl hydrocarbon receptor

SUPPLEMENTARY MATERIALS

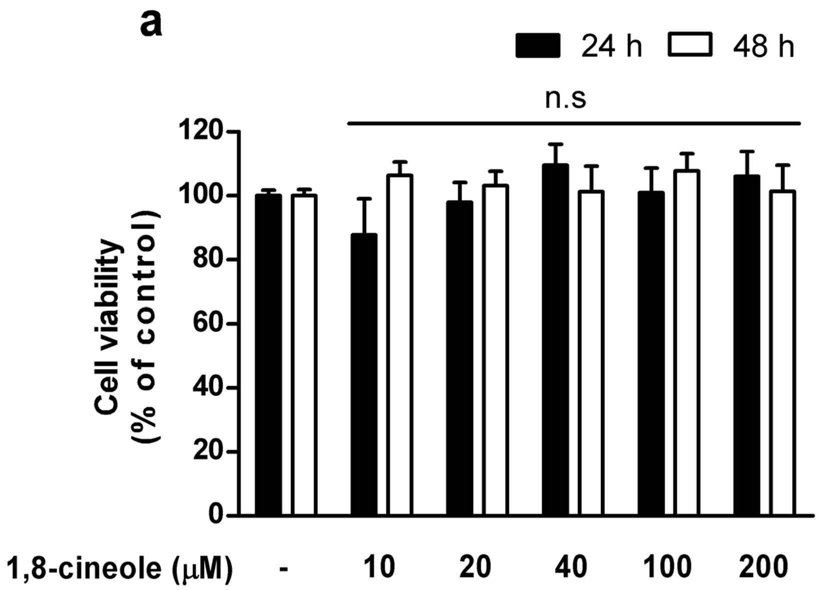

Supplementary Figure 1: Effect of 1,8-cineole on HaCaT cell viability. 1,8-cineole exhibits no cytotoxicity up to 200 μM in HaCaT cells. Cell viability was measured by MTS assay as described in 'Materials and Methods' section. Data are represented as the means ± SD (n = 3).

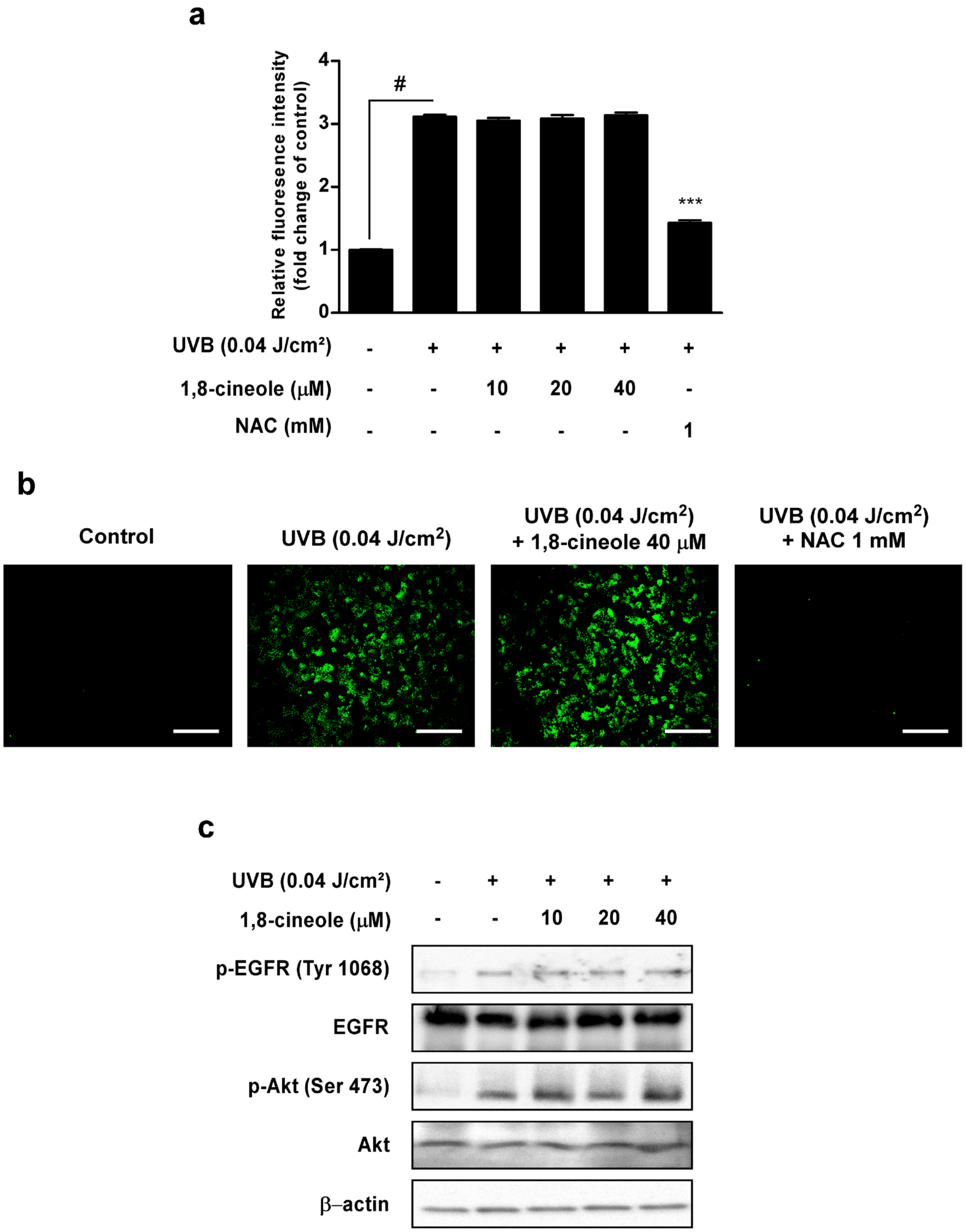

Supplementary Figure 2: Effect of 1,8-cineole on UVB-induced intracellular ROS generation and EGFR/Akt signaling pathway activation in HaCaT cells. (a) 1,8-cineole does not inhibit UVB-induced intracellular ROS generation. Cells were pretreated with 1,8-cineole and H_2-DCFDA for 1 hour before UVB irradiation. Following UVB treatment, intracellular ROS levels were immediately analyzed by fluorometer and represented as mean fluorescence intensity (MFI) units. NAC was used as a positive control. Data are presented as the means ± SD (n = 3). The hash symbol (#) indicates a significant difference between the control and the UVB-treated group; the asterisk symbol (***) indicates a significant difference ($p < 0.001$) between the UVB-treated group and the NAC-treated groups. Intracellular ROS generation was visualized by fluorescence microscopy. Scale bar, 100 μm.

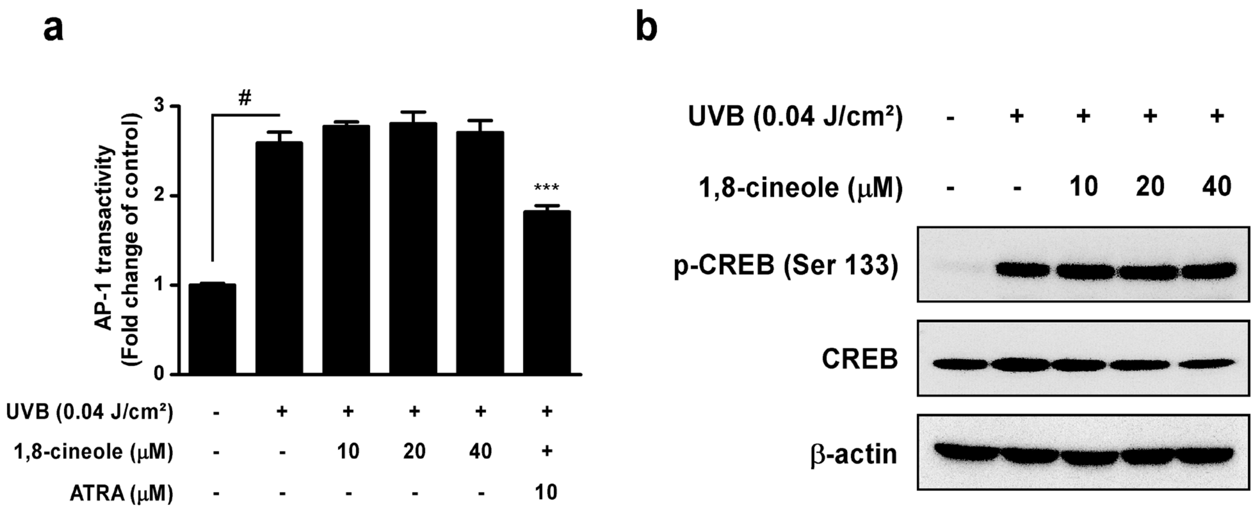

Supplementary Figure 3: Effect of 1,8-cineole on UVB-induced AP-1 transactivity and phosphorylation of CREB in HaCaT cells. (a) 1,8-cineole does not suppress UVB-induced AP-1 transactivation in HaCaT cells. The AP-1 luciferase assay is described in the 'Materials and Methods'. Results are shown as mean values ± S.D (n = 3). The symbol (#) indicates a significant difference (P < 0.05) between the control group and the UVB-irradiated group. The asterisks (***) indicate a significant difference (P < 0.001) between groups treated with UVB irradiation and all-*trans* retinoic acid (ATRA, positive control) and the group treated with UVB alone. **(b)** 1,8-cineole does not inhibit UVB-induced phosphorylation of CREB in HaCaT cells. Cells were pre-treated with 1,8-cineole at the indicated concentrations for 1 hour, irradiated with UVB, and then harvested after 30 min. Protein phosphorylation levels were detected by Western blotting.
